# 4-Methyl-*N*-(1-methyl-1*H*-indazol-5-yl)benzene­sulfonamide

**DOI:** 10.1107/S1600536813023398

**Published:** 2013-08-23

**Authors:** Hakima Chicha, Bassou Oulemda, El Mostapha Rakib, Mohamed Saadi, Lahcen El Ammari

**Affiliations:** a Laboratoire de Chimie Organique et Analytique, Université Sultan Moulay Slimane, Faculté des Sciences et Techniques, Béni-Mellal, BP 523, Morocco; b Laboratoire de Chimie du Solide Appliquée, Faculté des Sciences, Université Mohammed V-Agdal, Avenue Ibn Battouta, BP 1014, Rabat, Morocco

## Abstract

In the title compound, C_15_H_15_N_3_O_2_S, the fused ring system is close to planar, the largest deviation from the mean plane being 0.030 (2) Å, and makes a dihedral angle of 48.84 (9)° with the benzene ring belonging to the methyl­benzene­sulfonamide moiety. In the crystal, mol­ecules are ­connected through N—H⋯N hydrogen bonds and weak C—H⋯O contacts, forming a two-dimensional network parallel to (001).

## Related literature

For the pharmacological activity of sulfonamide derivatives, see: Bouissane *et al.* (2006 ▶); Mustafa *et al.* (2012 ▶); Lopez *et al.* (2010 ▶). For similar compounds, see: Abbassi *et al.* (2012 ▶, 2013 ▶).
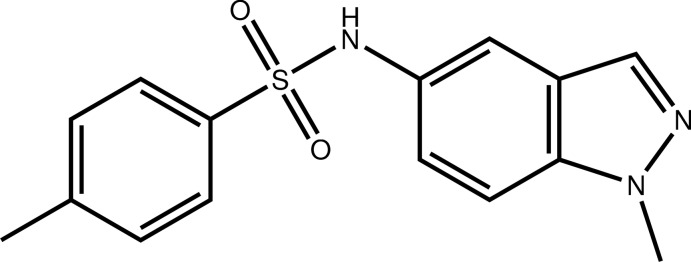


## Experimental

### 

#### Crystal data

C_15_H_15_N_3_O_2_S
*M* *_r_* = 301.36
Monoclinic, 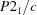

*a* = 8.0026 (3) Å
*b* = 12.8195 (4) Å
*c* = 14.1321 (4) Å
β = 91.602 (2)°
*V* = 1449.24 (8) Å^3^
*Z* = 4
Mo *K*α radiation
μ = 0.23 mm^−1^
*T* = 296 K
0.43 × 0.36 × 0.28 mm

#### Data collection

Bruker X8 APEX diffractometer
Absorption correction: multi-scan (*SADABS*; Bruker, 2009 ▶) *T*_min_ = 0.960, *T*_max_ = 0.992
17896 measured reflections
4048 independent reflections
2703 reflections with *I* > 2σ(*I*)
*R*_int_ = 0.047

#### Refinement

*R*[*F* ^2^ > 2σ(*F* ^2^)] = 0.046
*wR*(*F* ^2^) = 0.134
*S* = 1.02
4048 reflections
190 parameters
H-atom parameters constrained
Δρ_max_ = 0.25 e Å^−3^
Δρ_min_ = −0.32 e Å^−3^

### 

Data collection: *APEX2* (Bruker, 2009 ▶); cell refinement: *SAINT* (Bruker, 2009 ▶); data reduction: *SAINT*; program(s) used to solve structure: *SHELXS97* (Sheldrick, 2008 ▶); program(s) used to refine structure: *SHELXL97* (Sheldrick, 2008 ▶); molecular graphics: *ORTEP-3 for Windows* (Farrugia, 2012 ▶); software used to prepare material for publication: *PLATON* (Spek, 2009 ▶) and *publCIF* (Westrip, 2010 ▶).

## Supplementary Material

Crystal structure: contains datablock(s) I. DOI: 10.1107/S1600536813023398/bh2482sup1.cif

Structure factors: contains datablock(s) I. DOI: 10.1107/S1600536813023398/bh2482Isup2.hkl

Supplementary material file. DOI: 10.1107/S1600536813023398/bh2482Isup3.cml

Additional supplementary materials:  crystallographic information; 3D view; checkCIF report

## Figures and Tables

**Table 1:** Hydrogen-bond geometry (Å, °)

*D*—H⋯*A*	*D*—H	H⋯*A*	*D*⋯*A*	*D*—H⋯*A*
N1—H1⋯N2^i^	0.88	2.21	3.065 (2)	166
C3—H3⋯O2^ii^	0.93	2.53	3.277 (2)	137

Symmetry codes: (i) 
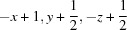
; (ii) 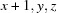.

**Fig. 1.**
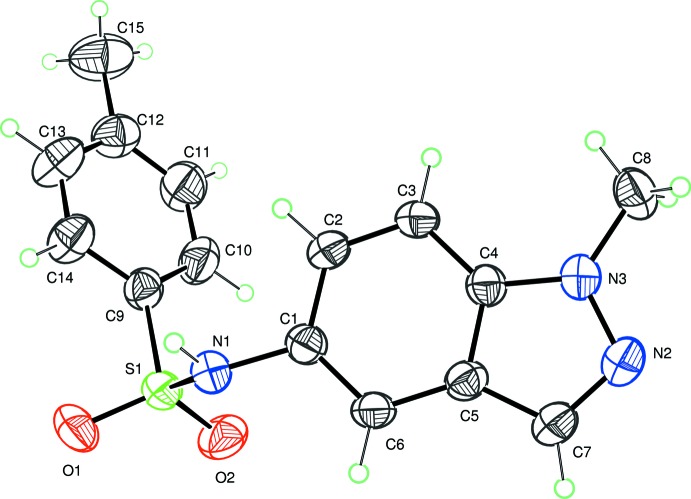
Molecular structure of the title compound with displacement ellipsoids for non-H atoms drawn at the 50% probability level. H atoms are represented as small circles.

**Fig. 2.**
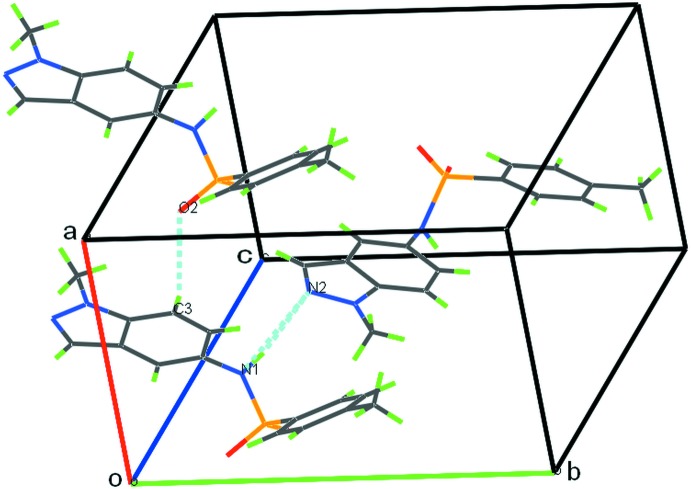
Partial crystal packing for the title compound showing C3—H3···O2 and N1—H1···N2 hydrogen bonds as blue dashed lines.

## supplementary crystallographic information

### 1. Comment

Sulfonamide derivatives are well known pharmaceutical agents since this group has been the main functional part of many drug structures, due to stability and tolerance in human beings. These compounds exhibit a wide range of biological activities, such as anticancer, anti-inflammatory, and antiviral functions (Bouissane *et al.*, 2006; Mustafa *et al.*, 2012; Lopez *et al.*, 2010). The present work is a continuation of the investigation on sulfonamide derivatives published recently by our team (Abbassi *et al.*, 2012, 2013).

The molecule of 4-methyl-*N*-(1-methyl-1*H*-indazol-5-yl)-benzenesulfonamide is built up from two fused five- and six-membered rings (N2/N3/C1 to C7) linked to the benzenesulfonamide group, as shown in Fig. 1. The fused rings system is almost planar, with the maximum deviation of 0.030 (2) Å arising from atom C1. Moreover, the dihedral angle between the indazole system and the plan through the atoms forming the benzene ring (C9 to C14) is 48.84 (9)°.

In the crystal, the molecules are interconnected through C3—H3···O2*^ii^* weak contacts and N1—H1···N2*^i^* hydrogen bonds, forming a two-dimensional network (Fig. 2 and Table 2; symmetry codes: (*i*) -*x* + 1, *y* + 1/2, -*z* + 1/2; (*ii*) *x* + 1, *y*, *z*).

### 2. Experimental

A mixture of 1-methyl-5-nitroindazole (1.22 mmol) and anhydrous SnCl_2_ (1.1 g, 6.1 mmol) in 25 ml of absolute ethanol was heated at 333 K for 6 h. After reduction, the starting material disappeared, and the solution was allowed to cool down. The pH was made slightly basic (pH 7–8) by addition of 5% aqueous potassium bicarbonate before extraction with ethyl acetate. The organic phase was washed with brine and dried over magnesium sulfate. The solvent was removed to afford the amine, which was immediately dissolved in pyridine (5 ml) and then reacted with 4-methylbenzenesulfonyl chloride (1.25 mmol) at room temperature for 24 h. After the reaction mixture was concentrated *in vacuo*, the resulting residue was purified by flash chromatography (eluted with ethyl acetate/hexane 1:9). The title compound was recrystallized from acetone.

### 3. Refinement

H atoms were located in a difference map, but C-bound H atoms were placed in idealized positions and treated as riding, with C—H = 0.96 Å, and C—H = 0.93 Å for methyl and aromatic CH, respectively. Atom H1 was first refined freely, and then fixed (N1—H1 = 0.8759 Å). All H atoms were refined with isotropic displacement parameters fixed as *U*_iso_(H) = 1.2*U*_eq_(C-aromatic, NH) or *U*_iso_(H) = 1.5*U*_eq_(*C*-methyl).

### Crystal data

**Table:**

C_15_H_15_N_3_O_2_S	*F*(000) = 632
*M**_r_* = 301.36	*D*_x_ = 1.381 Mg m^−^^3^
Monoclinic, *P*2_1_/*c*	Mo *K*α radiation, λ = 0.71073 Å
Hall symbol: -P 2ybc	Cell parameters from 4048 reflections
*a* = 8.0026 (3) Å	θ = 2.9–29.6°
*b* = 12.8195 (4) Å	µ = 0.23 mm^−^^1^
*c* = 14.1321 (4) Å	*T* = 296 K
β = 91.602 (2)°	Block, colourless
*V* = 1449.24 (8) Å^3^	0.43 × 0.36 × 0.28 mm
*Z* = 4	

### Data collection

**Table:**

Bruker X8 APEX diffractometer	4048 independent reflections
Radiation source: fine-focus sealed tube	2703 reflections with *I* > 2σ(*I*)
Graphite monochromator	*R*_int_ = 0.047
φ and ω scans	θ_max_ = 29.6°, θ_min_ = 2.9°
Absorption correction: multi-scan (*SADABS*; Bruker, 2009)	*h* = −11→11
*T*_min_ = 0.960, *T*_max_ = 0.992	*k* = −17→17
17896 measured reflections	*l* = −19→19

### Refinement

**Table:**

Refinement on *F*^2^	Primary atom site location: structure-invariant direct methods
Least-squares matrix: full	Secondary atom site location: difference Fourier map
*R*[*F*^2^ > 2σ(*F*^2^)] = 0.046	Hydrogen site location: inferred from neighbouring sites
*wR*(*F*^2^) = 0.134	H-atom parameters constrained
*S* = 1.02	*w* = 1/[σ^2^(*F*_o_^2^) + (0.0633*P*)^2^ + 0.2552*P*] where *P* = (*F*_o_^2^ + 2*F*_c_^2^)/3
4048 reflections	(Δ/σ)_max_ < 0.001
190 parameters	Δρ_max_ = 0.25 e Å^−^^3^
0 restraints	Δρ_min_ = −0.32 e Å^−^^3^
0 constraints	

### Fractional atomic coordinates and isotropic or equivalent isotropic displacement parameters (Å^2^)

**Table:**

	*x*	*y*	*z*	*U*_iso_*/*U*_eq_	
C1	0.31920 (19)	0.13913 (14)	0.21183 (12)	0.0348 (4)	
C2	0.4588 (2)	0.18535 (14)	0.16945 (13)	0.0386 (4)	
H2	0.4693	0.2576	0.1706	0.046*	
C3	0.5789 (2)	0.12762 (14)	0.12688 (13)	0.0381 (4)	
H3	0.6709	0.1587	0.0997	0.046*	
C4	0.55641 (19)	0.01961 (13)	0.12629 (12)	0.0336 (4)	
C5	0.4161 (2)	−0.02812 (13)	0.16553 (12)	0.0352 (4)	
C6	0.2952 (2)	0.03317 (13)	0.20985 (13)	0.0361 (4)	
H6	0.2022	0.0029	0.2368	0.043*	
C7	0.4424 (2)	−0.13606 (15)	0.15117 (14)	0.0448 (4)	
H7	0.3685	−0.1880	0.1690	0.054*	
C8	0.8127 (2)	−0.04980 (17)	0.04713 (14)	0.0486 (5)	
H8A	0.8953	−0.0912	0.0803	0.073*	
H8B	0.8477	0.0218	0.0470	0.073*	
H8C	0.8000	−0.0743	−0.0169	0.073*	
C9	0.1242 (2)	0.36208 (14)	0.13664 (13)	0.0392 (4)	
C10	0.1538 (3)	0.35014 (17)	0.04145 (16)	0.0561 (5)	
H10	0.1315	0.2868	0.0116	0.067*	
C11	0.2163 (3)	0.43217 (19)	−0.00880 (16)	0.0616 (6)	
H11	0.2356	0.4234	−0.0729	0.074*	
C12	0.2515 (3)	0.52718 (17)	0.03268 (16)	0.0523 (5)	
C13	0.2195 (3)	0.53815 (18)	0.12835 (17)	0.0617 (6)	
H13	0.2415	0.6016	0.1580	0.074*	
C14	0.1560 (3)	0.45732 (16)	0.17998 (15)	0.0518 (5)	
H14	0.1345	0.4664	0.2438	0.062*	
C15	0.3195 (3)	0.6169 (2)	−0.0239 (2)	0.0802 (8)	
H15A	0.3339	0.5951	−0.0881	0.120*	
H15B	0.4253	0.6383	0.0032	0.120*	
H15C	0.2425	0.6742	−0.0227	0.120*	
N1	0.20248 (17)	0.20314 (12)	0.26172 (11)	0.0393 (3)	
H1	0.2468	0.2458	0.3038	0.047*	
N2	0.5857 (2)	−0.15374 (12)	0.10917 (12)	0.0471 (4)	
N3	0.65480 (18)	−0.05848 (12)	0.09380 (11)	0.0391 (3)	
O1	−0.05599 (17)	0.30214 (12)	0.27498 (12)	0.0624 (4)	
O2	−0.02085 (16)	0.18164 (11)	0.13971 (11)	0.0572 (4)	
S1	0.04597 (5)	0.25800 (4)	0.20396 (4)	0.04273 (16)	

### Atomic displacement parameters (Å^2^)

**Table:**

	*U*^11^	*U*^22^	*U*^33^	*U*^12^	*U*^13^	*U*^23^
C1	0.0324 (7)	0.0340 (9)	0.0381 (9)	0.0007 (6)	0.0028 (6)	0.0028 (7)
C2	0.0387 (8)	0.0284 (9)	0.0491 (11)	−0.0039 (7)	0.0045 (7)	0.0023 (8)
C3	0.0336 (8)	0.0340 (10)	0.0469 (10)	−0.0041 (7)	0.0073 (7)	0.0040 (8)
C4	0.0314 (7)	0.0335 (9)	0.0358 (9)	0.0026 (6)	−0.0003 (6)	0.0017 (7)
C5	0.0345 (8)	0.0307 (9)	0.0404 (10)	−0.0014 (6)	−0.0010 (7)	0.0040 (7)
C6	0.0312 (7)	0.0330 (9)	0.0441 (10)	−0.0039 (6)	0.0041 (7)	0.0060 (8)
C7	0.0462 (10)	0.0309 (10)	0.0574 (12)	−0.0021 (7)	0.0041 (8)	0.0041 (9)
C8	0.0448 (10)	0.0532 (13)	0.0483 (11)	0.0088 (8)	0.0113 (8)	−0.0031 (10)
C9	0.0359 (8)	0.0353 (10)	0.0461 (10)	0.0035 (7)	−0.0006 (7)	−0.0016 (8)
C10	0.0714 (13)	0.0429 (12)	0.0544 (13)	−0.0043 (10)	0.0093 (10)	−0.0120 (10)
C11	0.0802 (16)	0.0564 (15)	0.0488 (13)	−0.0019 (12)	0.0119 (11)	0.0001 (11)
C12	0.0515 (11)	0.0466 (13)	0.0586 (14)	−0.0009 (9)	−0.0033 (9)	0.0106 (10)
C13	0.0813 (16)	0.0415 (13)	0.0619 (15)	−0.0138 (11)	−0.0080 (12)	−0.0026 (11)
C14	0.0674 (13)	0.0420 (12)	0.0456 (11)	−0.0050 (9)	−0.0026 (9)	−0.0027 (9)
C15	0.0803 (17)	0.0709 (18)	0.0893 (19)	−0.0147 (14)	−0.0032 (14)	0.0316 (16)
N1	0.0382 (7)	0.0352 (8)	0.0448 (9)	0.0009 (6)	0.0081 (6)	−0.0017 (7)
N2	0.0510 (9)	0.0323 (9)	0.0582 (10)	0.0030 (7)	0.0029 (7)	−0.0006 (8)
N3	0.0386 (7)	0.0352 (9)	0.0436 (9)	0.0038 (6)	0.0033 (6)	−0.0012 (7)
O1	0.0494 (8)	0.0546 (10)	0.0848 (11)	0.0102 (6)	0.0318 (8)	0.0029 (8)
O2	0.0403 (7)	0.0446 (8)	0.0862 (11)	−0.0080 (6)	−0.0079 (7)	−0.0064 (8)
S1	0.0311 (2)	0.0361 (3)	0.0614 (3)	0.00089 (16)	0.00821 (18)	−0.0010 (2)

### Geometric parameters (Å, º)

**Table:**

C1—C6	1.372 (2)	C9—C14	1.387 (3)
C1—C2	1.412 (2)	C9—S1	1.7635 (19)
C1—N1	1.442 (2)	C10—C11	1.371 (3)
C2—C3	1.366 (2)	C10—H10	0.9300
C2—H2	0.9300	C11—C12	1.377 (3)
C3—C4	1.396 (2)	C11—H11	0.9300
C3—H3	0.9300	C12—C13	1.390 (3)
C4—N3	1.361 (2)	C12—C15	1.510 (3)
C4—C5	1.406 (2)	C13—C14	1.373 (3)
C5—C6	1.407 (2)	C13—H13	0.9300
C5—C7	1.415 (3)	C14—H14	0.9300
C6—H6	0.9300	C15—H15A	0.9600
C7—N2	1.325 (2)	C15—H15B	0.9600
C7—H7	0.9300	C15—H15C	0.9600
C8—N3	1.446 (2)	N1—S1	1.6348 (15)
C8—H8A	0.9600	N1—H1	0.8759
C8—H8B	0.9600	N2—N3	1.361 (2)
C8—H8C	0.9600	O1—S1	1.4280 (14)
C9—C10	1.381 (3)	O2—S1	1.4288 (15)
			
C6—C1—C2	121.25 (15)	C9—C10—H10	120.2
C6—C1—N1	118.75 (14)	C10—C11—C12	122.1 (2)
C2—C1—N1	119.96 (15)	C10—C11—H11	119.0
C3—C2—C1	122.27 (16)	C12—C11—H11	119.0
C3—C2—H2	118.9	C11—C12—C13	117.5 (2)
C1—C2—H2	118.9	C11—C12—C15	121.4 (2)
C2—C3—C4	116.61 (15)	C13—C12—C15	121.1 (2)
C2—C3—H3	121.7	C14—C13—C12	121.4 (2)
C4—C3—H3	121.7	C14—C13—H13	119.3
N3—C4—C3	130.97 (16)	C12—C13—H13	119.3
N3—C4—C5	106.78 (15)	C13—C14—C9	119.7 (2)
C3—C4—C5	122.23 (15)	C13—C14—H14	120.1
C4—C5—C6	119.96 (16)	C9—C14—H14	120.1
C4—C5—C7	104.22 (15)	C12—C15—H15A	109.5
C6—C5—C7	135.76 (16)	C12—C15—H15B	109.5
C1—C6—C5	117.65 (15)	H15A—C15—H15B	109.5
C1—C6—H6	121.2	C12—C15—H15C	109.5
C5—C6—H6	121.2	H15A—C15—H15C	109.5
N2—C7—C5	111.41 (16)	H15B—C15—H15C	109.5
N2—C7—H7	124.3	C1—N1—S1	119.89 (12)
C5—C7—H7	124.3	C1—N1—H1	115.6
N3—C8—H8A	109.5	S1—N1—H1	111.2
N3—C8—H8B	109.5	C7—N2—N3	106.19 (15)
H8A—C8—H8B	109.5	N2—N3—C4	111.38 (14)
N3—C8—H8C	109.5	N2—N3—C8	120.41 (15)
H8A—C8—H8C	109.5	C4—N3—C8	128.20 (16)
H8B—C8—H8C	109.5	O1—S1—O2	120.46 (9)
C10—C9—C14	119.56 (19)	O1—S1—N1	105.33 (9)
C10—C9—S1	120.99 (15)	O2—S1—N1	106.88 (8)
C14—C9—S1	119.44 (15)	O1—S1—C9	107.32 (9)
C11—C10—C9	119.6 (2)	O2—S1—C9	107.92 (9)
C11—C10—H10	120.2	N1—S1—C9	108.45 (8)
			
C6—C1—C2—C3	−1.9 (3)	C12—C13—C14—C9	0.6 (4)
N1—C1—C2—C3	175.55 (16)	C10—C9—C14—C13	−1.2 (3)
C1—C2—C3—C4	0.6 (3)	S1—C9—C14—C13	178.93 (17)
C2—C3—C4—N3	−176.89 (18)	C6—C1—N1—S1	−95.37 (17)
C2—C3—C4—C5	1.3 (3)	C2—C1—N1—S1	87.17 (18)
N3—C4—C5—C6	176.56 (15)	C5—C7—N2—N3	−0.9 (2)
C3—C4—C5—C6	−2.0 (3)	C7—N2—N3—C4	0.3 (2)
N3—C4—C5—C7	−0.94 (19)	C7—N2—N3—C8	−179.02 (17)
C3—C4—C5—C7	−179.50 (16)	C3—C4—N3—N2	178.82 (18)
C2—C1—C6—C5	1.1 (3)	C5—C4—N3—N2	0.4 (2)
N1—C1—C6—C5	−176.33 (15)	C3—C4—N3—C8	−1.9 (3)
C4—C5—C6—C1	0.7 (3)	C5—C4—N3—C8	179.70 (17)
C7—C5—C6—C1	177.3 (2)	C1—N1—S1—O1	171.99 (13)
C4—C5—C7—N2	1.2 (2)	C1—N1—S1—O2	42.75 (15)
C6—C5—C7—N2	−175.7 (2)	C1—N1—S1—C9	−73.37 (14)
C14—C9—C10—C11	0.8 (3)	C10—C9—S1—O1	−147.78 (17)
S1—C9—C10—C11	−179.30 (18)	C14—C9—S1—O1	32.12 (18)
C9—C10—C11—C12	0.2 (4)	C10—C9—S1—O2	−16.55 (18)
C10—C11—C12—C13	−0.8 (4)	C14—C9—S1—O2	163.35 (15)
C10—C11—C12—C15	−179.7 (2)	C10—C9—S1—N1	98.89 (17)
C11—C12—C13—C14	0.4 (4)	C14—C9—S1—N1	−81.21 (17)
C15—C12—C13—C14	179.3 (2)		

### Hydrogen-bond geometry (Å, º)

**Table:**

*D*—H···*A*	*D*—H	H···*A*	*D*···*A*	*D*—H···*A*
N1—H1···N2^i^	0.88	2.21	3.065 (2)	166
C3—H3···O2^ii^	0.93	2.53	3.277 (2)	137

Symmetry codes: (i) −*x*+1, *y*+1/2, −*z*+1/2; (ii) *x*+1, *y*, *z*.
